# Sexual behavior according to undergraduate students: perspective of cross-cultural nursing and intersectional framing

**DOI:** 10.1590/0034-7167-2022-0786

**Published:** 2023-12-04

**Authors:** Laércio Deleon de Melo, Thelma Spindola, Cristina Arreguy-Sena, Paula Krempser, Juliana de Lima Brandão, Cristiane Maria Amorim Costa, Felipe Eduardo Taroco, Paulo Ferreira Pinto

**Affiliations:** I Universidade do Estado do Rio de Janeiro. Rio de Janeiro, Rio de Janeiro, Brazil; II Universidade Federal de Juiz de Fora. Juiz de Fora, Minas Gerais, Brazil; III Centro Universitário Estácio Juiz de Fora. Juiz de Fora, Minas Gerais, Brazil; IV Universidade Norte do Paraná. Londrina, Paraná, Brazil

**Keywords:** Young Adult, Sexual Behavior, Cross-Cultural Nursing, Intersectional Framing, Sexually Transmitted Infections, Adulto Joven, Conducta Sexual, Marco Interseccional, Enfermería Transcultural, Enfermedades de Transmisión Sexual, Adulto Jovem, Comportamento Sexual, Enfermagem Transcultural, Enquadramento Interseccional, Infecções Sexualmente Transmissíveis

## Abstract

**Objective:**

to discuss undergraduate students’ sexual behavior from the perspective of social markers and cross-cultural care proposed by Madeleine Leininger.

**Methods:**

descriptive-exploratory qualitative research, with a theoretical-philosophical foundation in the Transcultural Theory. Convenience sample was composed of 57 young people from two universities in Rio de Janeiro. The focus groups’ content were analyzed lexically using the IRAMUTEQ software.

**Results:**

four classes emerged: Young people’s sexual scripts: between the fear of an unplanned pregnancy and the risk of exposure to sexually transmitted infections; Affective relationships: trust in steady sexual partners, apparent sense of security and disuse of condoms; Sexual practices, gender and cultural determinants: distinction in men’s and women’s role; Sexual partnerships, negotiation of condom use and vulnerability to sexually transmitted infections.

**Final considerations:**

challenges are perceived for the attention to undergraduate students’ sexual health, who verbalized risky sexual behaviors due to sociocultural vulnerabilities.

## RESUMO

**Objetivo:**

discutir os comportamentos sexuais de jovens universitários na perspectiva dos marcadores sociais e do cuidado transcultural proposto por Madeleine Leininger.

**Métodos:**

pesquisa qualitativa descritiva-exploratória, com alicerce teórico-filosófico na Teoria Transcultural. Amostragem por conveniência foi composta por 57 jovens de duas universidades cariocas. Os conteúdos dos grupos focais foram analisados lexicalmente pelo *software* IRAMUTEQ.

**Resultados:**

emergiram quatro classes: *Roteiros sexuais de jovens: entre o receio de uma gestação não planejada e o risco da exposição às infecções sexualmente transmissíveis; Relacionamentos afetivos: a confiança em parcerias sexuais fixas, aparente sensação de segurança e o desuso dos preservativos; Práticas sexuais, gênero e determinantes culturais: distinção nos papéis de homens e mulheres; Parcerias sexuais, negociação do uso do preservativo e a vulnerabilidade às infecções sexualmente transmissíveis*.

**Considerações finais:**

percebem-se desafios para atenção à saúde sexual dos jovens universitários, que verbalizaram comportamentos sexuais de risco em função de vulnerabilidades socioculturais.

## RESUMEN

**Objetivo:**

discutir el comportamiento sexual de estudiantes universitarios en la perspectiva de los marcadores sociales y el cuidado transcultural propuesto por Madeleine Leininger.

**Métodos:**

investigación cualitativa descriptiva-exploratoria, con fundamento teórico-filosófico en la Teoría Transcultural. La muestra de conveniencia fue compuesta por 57 jóvenes de dos universidades de Río de Janeiro. Los contenidos de los grupos focales se analizaron léxicamente mediante el software IRAMUTEQ.

**Resultados:**

surgieron cuatro clases: Guiones sexuales de jóvenes: entre el miedo a un embarazo no planeado y el riesgo de exposición A INFECCIONES DE TRANSMISIÓN SEXUAL; Relaciones afectivas: confianza en parejas sexuales estables, aparente sensación de seguridad y desuso de preservativos; Prácticas sexuales, género y determinantes culturales: distinción en los roles de hombres y mujeres y; Parejas sexuales, negociación del uso del condón y vulnerabilidad a las infecciones de transmisión sexual.

**Consideraciones finales:**

se perciben desafíos para la atención a la salud sexual de estudiantes universitarios, quienes verbalizaron conductas sexuales de riesgo, debido a vulnerabilidades socioculturales.

## INTRODUCTION

The World Health Organization (WHO) estimated a total of incident cases of curable Sexually Transmitted Infections (STIs) at 376.4 million people. It is believed that there is a daily worldwide occurrence of more than one million new people infected by some type of STI. Approximately 500 million are infected annually by some curable STI, in addition to the high rates of incurable infections^(1)^. These STIs have multiple unfavorable impacts on people’s lives. The high rates of contamination, transmission and morbidity and mortality raise concerns about global health^(1-2)^. STIs gained greater visibility from the 1980s onwards with the emergence of the Acquired Immunodeficiency Syndrome (AIDS), but they are still a public health problem that requires actions to control sociocultural determinants^(3-4)^.

It should be noted that gonorrhea, human papillomavirus (HPV), syphilis, Human Immunodeficiency Virus (HIV), herpes and chlamydia are the most prevalent STIs among young people. Socioeconomic damage is increasing, due to the greater number of people infected in many countries, serious consequences for sexual, reproductive and maternal-fetal health, high potential for transmissibility and contagion, and strong correlation with forms of expression of sexuality and sexual practices^(1-2,4)^.

The issue of acquiring STI coexists in social thinking from the moment sexual practices are initiated. In this context, a scientific gap was identified, making it timely to re-read the current sociocultural determinants and sexual behaviors adopted by undergraduate students, in order to contemplate the peculiarities of the academic context marked by diversity, freedom, discoveries and flexibility in expressing individualities and behavioral characteristics^(4)^. Moreover, due to the natural complexity of issues involving young undergraduate students’ sexual health, it is necessary to contemplate culture in terms of health care, preserving the subjectivities of care subjects^(3,5)^. In this regard, this perspective is related to Leininger’s Transcultural Nursing Theory, which is anchored in the planning and implementation of (self)care actions, which is anchored in the planning and implementation of (self)care actions that consider the cultural contribution of the person being cared for, in order to guarantee congruent care to multiple health demands^(5)^.

Added value to this investigation is the fact that the results are reflected and discussed from the perspective of a theoretical-philosophical nursing framework, which includes social markers, with emphasis on understanding participants’ sexual behaviors. This is because the university environment corroborates the emergence of variables and constraints of sexual behavior in favor of the adoption of STI prevention practices and/or risky sexual behaviors (RSB) as characteristic markers of the university phase that reveal vulnerabilities linked to sociocultural determinants^(4)^.

The epidemiological relevance of the theme and the importance of the possible contributions of this investigation to the fields of nursing, health and education are highlighted as an axis of care that requires interdisciplinary interventions, with a focus on disease prevention actions, health promotion, early diagnosis and syndromic approach to STIs. This investigation is part of a thematic call “Good practices and challenges in health care with groups living in vulnerable situations”, with the guiding question being: how are the sexual behaviors adopted by undergraduate students interrelated with social markers and cross-cultural care from Leininger’s perspective?

## OBJECTIVE

To discuss undergraduate students’ sexual behavior from the perspective of social markers and cross-cultural care proposed by Madeleine Leininger.

## METHODS

### Ethical aspects

The matrix investigation “*Sexualidade e vulnerabilidade dos jovens em tempos de infecções sexualmente transmissíveis*” was approved by the Research Ethics Committee (REC) of the *Universidade do Estado do Rio de Janeiro* (UERJ) for release of data collection at the two Higher Education Institutions (HEIs) in Rio de Janeiro, whose consubstantiated opinion is attached to this submission. All guidelines and ethical norms for research involving national and international human beings were complied with. Participant acquiescence was expressed by signing, in writing, the Informed Consent Form (ICF), post-informed, in person, as a step that preceded the data collection.

### Theoretical-methodological framework

In the search for a nursing framework consistent with the intended sociocultural approach, it was possible to identify that Madeleine Leininger’s Transcultural Nursing Theory is able to provide the theoretical-philosophical foundation structuring the reflective and explanatory processes of this investigation. In interpersonal interactions and in the multiple contexts of human health care, diversified professional actions and individual creativity must be used, in order to preserve, negotiate or re-standardize care, seeking a sociocultural congruence^(5)^.

The Transcultural Nursing Theory can be conceived as the study of beliefs, values and nursing care practices, as perceived and cognitively known by a given culture, through its direct experience, through the expression of beliefs and value systems^(3-4)^. Thus, the importance that sociocultural forces exert on human beings is valued and, consequently, affect the care process. In this conception, the absence of the cultural factor in care results in care that is disconnected from a person’s reality, and this incongruity in relation to values and beliefs may cause signs of cultural conflicts, frustrations, stress and/or moral and ethical concerns^(5)^. Thus, the markers (gender/sex, class/income, ethnicity/race, religion, sexual orientation, generation/age) involved in the health-disease process must be considered in the study of sociocultural realities, and require an intersectional look^(6)^.

### Study design

This is a qualitative, descriptive and exploratory research, with the use of a deepening of the theme, in addition to aiming at understanding the investigated phenomenon^(7)^. To this end, the COnsolidated criteria for REporting Qualitative research (COREQ) protocol was respected, which safeguards the quality of qualitative studies in a checklist^(8)^.

### Methodological procedures

Participants were recruited through individual contact at the research institutions. On that occasion, there was an explanation by the researchers regarding the objectives, purposes, risks and benefits of participating in the study, with the clarification of possible doubts presented as well as the rights of participants and duties of the researcher.

The data collection instrument contained a thematic script intended to support the focus groups (FG), prepared by the researcher responsible for the matrix investigation, in which students’ reports on sexual behavior were explored. Thus, six themes were selected, namely: the young person and his characterization; sexuality; sexual behavior and gender; STI; vulnerability to STIs; and sexual health care/health education.

### Study setting

Data were collected from two HEIs in the city of Rio de Janeiro (RJ), Brazil, one public and one private university. The choice of these settings was justified by the investigative possibilities sought with the approximations of the profile of undergraduate students, from the public and private systems, with regard to the construction of sexual behaviors.

### Data source

Undergraduate students aged between 18 and 29 years old participated in the study. To delimit the age group, the Brazilian Youth Statute was adopted as a reference (young people are people aged between 15-29 years)^(3)^. However, it was decided not to include students aged ≤17 years, for ethical and legal reasons (requirement of consent of the person responsible and the assent form).

We included students aged ≥18 years, regularly enrolled in one of the undergraduate courses offered by the institution and having started their sexual life. We excluded students who were absent from the data collection field, with enrollment or sick leave.

### Data collection and organization

Data were collected during the 2018 school periods, on the premises of the two HEIs. The FGs were carried out at previously scheduled times and dates, with audio recording on a Sony Px 240 - 4g Memo Digital Voice Mini Recorder, aiming at the detailed capture of information, guaranteeing the reliability of collected contents. The FG technique is a form of interview with groups made up of eight to 12 participants, based on communication and interaction, which aims to gather detailed information on a specific topic (proposed and presented by the researcher) for a group of selected participants^(7)^. The role of observer and secretary of records in all FGs was played by two collaborating master’s students in the collection stage.

The FG lasted between 90 and 120 minutes, and occurred without any kind of influence from the conceptions of the researchers involved^(7,9)^. At HEI-1, three meetings were held with ten participants each (n: 30). At HEI-2, the three meetings were made up of nine, eight and ten students, respectively (n: 27). Thus, a convenience sampling was adopted, consisting of 57 undergraduate students, according to theoretical consolidation criteria^(7)^.

### Data analysis

The contents were transcribed in full in Word for Windows 2016 from Microsoft^®^ Office and, subsequently, the corpus was formatted to use the *R Interface Pour les Analyzes Multidimensionnelles de Textes et de Questionnaires* (IRAMUTEQ), version 0.7 alpha 2, by lexical analysis. This software is a free and open source qualitative data manager, which allows performing classic textual statistical analysis, basic lexicographical analysis (word frequency calculation), multivariate analysis (similarity analysis and word cloud), group specificity research and Descending Hierarchical Classification (DHC)^(10)^. In this investigation, DHC was chosen, in which the text segments are divided and classified according to vocabulary from lemmatized words.

## RESULTS

**Figure 1:**
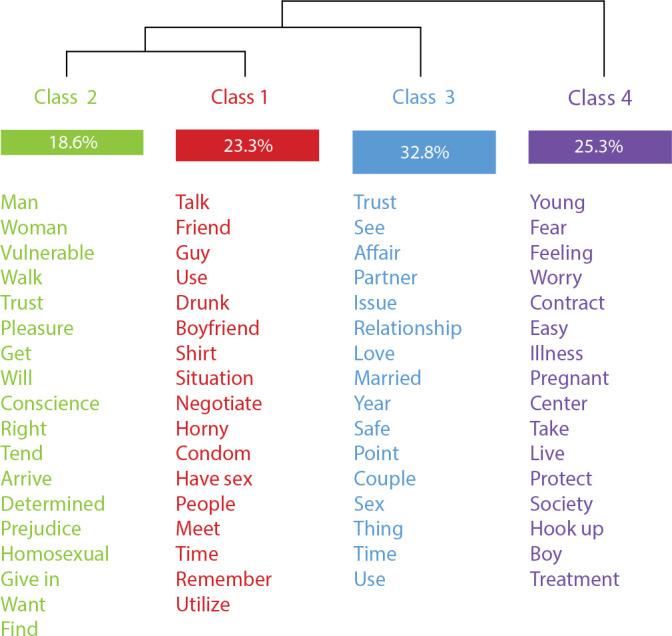
Dendrogram with the distribution of classes and semantic contents of sexual behaviors, Rio de Janeiro, Rio de Janeiro, Brazil, 2023

*Source: extracted from IRAMUTEQ, 2023.*

The 57 undergraduate students were mostly women (48%), aged between 18 and 24 years (82.7%), white skin color (51.4%), single (52.1%), heterosexual (84.9%). Thus, they lived with their parents (70.9%); did not work (58.0%); had income classified as low-upper class (23.8%); they considered themselves to be religious practitioners (62%) and of Catholic religious background (43.3%). In the lexical analysis, the FG corpus had an 81.09% use by IRAMUTEQ, which identified 312 elementary context units (ECU), distributed in four classes, from successive binary divisions of the corpus (Figure 1).

The corpus was initially divided into two axes. Axis 1 generated class 4. Axis 2 generated class 3, and, after further subdivision, formed classes 2 and 1. Thus, at the end of the cleavage process, the corpus was divided into four classes. Obeying the young people’s discursive content and with a view to favoring cluster analysis visualization, the classes were renamed. The most expressive words (x^2^), for each class, are shown in Figure 1.

### Class 4 - Young people’s sexual scripts - between the fear of an unplanned pregnancy and the risk of exposure to sexually transmissible infections

It consists of 64 ECU, which corresponds to 25.3% of the total classes, being the second largest class that emerged in the analysis process. The words associated with it translate the social thinking that modulates the behavior of undergraduate students, between the fear of an unplanned pregnancy and the risks of exposure to STIs.

*I see that people are more concerned with not getting pregnant than with not getting a disease. They worry more about taking a morning-after pill than using a condom.* (P07, female, heterosexual)

*There are different behaviors, there are those young people who are very radical, they live without responsibility, I can contract it or I cannot contract it. I know and have friends who like the feeling of having sex without a condom, not because of the fear of contracting an STI or not, but because it is more pleasurable. And if later there are consequences, then we go to the post and carry out treatment. But there are conscious young people who are aware of the diseases, STIs, the risks and are prevented. For others, that sense of danger is more enjoyable. I like this feeling that I can get infected, or I can have sex with someone who has HIV and not get infected. Sometimes, this feeling for some young people can be exciting.* (P14, female, bisexual)

*The fear is having a child and not a disease. Men always want to expose themselves.* (P33, heterosexual woman)

*The woman is more afraid of getting pregnant than of thinking about the disease.* (P49, woman, homosexual)

### Class 3 - Affective relationships: trust in steady sexual partners, the apparent sense of security and disuse of condoms

Class was composed of 83 ECU corresponding to 32.8% of the total of classes, the most significant being. The associated words represent the relational, affective and sexual contexts of undergraduate students, in which behaviors are determined by the degree of trust in sexual partnerships and the non-use of condoms when they feel safe.

*I believe that, throughout marriage, people end up giving in because of trust. There is also the question that you want to have skin-to-skin contact with your partner.* (P09, female, heterosexual)

*There is the issue of trust, it passes and people think, “I won’t use a condom, I’ll just take the contraceptive”. Will it do anything? It’s not just the pregnancy that matters.* (P12, female, heterosexual)

*I’m married, I’m used to having sex with only one, so I don’t need a condom.* (P15, male, homosexual)

*Some still ask and use the psychological and say, “If you love me, you won’t use a condom”.* (P32, male, homosexual)

*If you ask married people, they almost don’t use it because it’s a couple, there’s this issue of trust.* (P41, woman, heterosexual)

*In all relationships, we use a condom partially, but only that way, only at the end to prevent pregnancy. I even use a condom, but only to prevent pregnancy because I always trusted my boyfriend.* (P44, woman, heterosexual)

### Class 2 - Sexual practices, gender and cultural determinants: distinction in the roles of men and women

The class was composed of 47 ECU, which correspond to 18.58% of all classes (< analysis process class). The words associated with it translate into sexual practices and into the cultural determinants of undergraduate students regarding the distinction of roles according to gender.

*For women, there is domination over sex. For men, it is enough to say that they will not use a condom and, in their socially constructed mind, they are right and women cannot give an opinion.* (P03, woman, heterosexual)

*They also have the same rights as men. Nowadays, women also have sex with multiple partners.* (P10, male, heterosexual)

*Women expect a lot from men, they should wear a condom. The thought is that women cannot wear a condom.* (P12, woman, heterosexual)

*I don’t want to, that’s the rarest thing in the world, for a woman to say no. If you don’t know her, she ends up feeling more comfortable herself, because, in fact, women find it difficult to say, “I don’t want to without a condom”.* (P16, male, heterosexual)

*There are women who carry a male condom in their purse.* (P25, male, heterosexual)

*Men are more vulnerable, because they are more reckless and want to have sex without a condom.* (P32, male, homosexual)

*Women are more determined, they also have casual sex, assume that they feel pleasure and have a sexual need.* (P42, woman, heterosexual)

*In general, men are more vulnerable than women, both heterosexual and homosexual to these diseases.* (P45, male, homosexual)

*The man assumes the position of insisting on not using a condom and the woman assumes the position of being the one who should give in and not use it.* (P53, male, heterosexual)

### Class 1 - Sexual partnerships, negotiation of condom use and vulnerability to sexually transmitted infections

The class was composed of 59 ECU, which correspond to 23.3% of all classes (third most significant class of analysis). The associated words portray the types of sexual partnerships of undergraduate students, their vulnerabilities to STIs and the negotiation of condom use.

*Men will carry a condom in the same way that women can buy it and keep it hidden.* (P02, male, heterosexual)

*You are going to negotiate, but the guy is going to say, “I like condoms and I only use them”, and the same thing for women to say to me, “Just use them and want them that way, then let’s go, if you don’t want them, then patience”. Today there are women with a boyfriend or hookup for every day of the week, there is no longer that “I can’t” shyness. There are people who don’t use a condom or are drunk and don’t have a condom.* (P10, male, heterosexual)

*Most have sex without a condom. There are a lot of guys who say it bothers them.* (P24, male, heterosexual)

*It completely changes the perception of any situation, with alcohol, it’s more complicated, but I’ve had sex with the guy, like, the guy was very drunk and I couldn’t even put the condom on properly, that’s how it was.* (P35, woman, homosexual)

*My friends say they don’t have sex with a condom, because they say, “If the initiative comes from me, like I brought it, I have it, they’ll think I went out to have sex”.* (P42, woman, heterosexual)

*Women also carry condoms because men often say “I didn’t bring them” and try to force the relationship without you being able to say “But I brought them”.* (P54, woman, heterosexual)

## DISCUSSION

The sociodemographic characterization profile was similar to that found in other investigations with undergraduate students^(3,11-13)^. For young people, sexuality is presented in a field of experiences, communications, feelings, experiences and discoveries, in the construction of the ability to make decisions, their preferences and the certification of an individual identity. In the search for sexual practices’ autonomy, sexuality has been performed in a peculiar and individualized way^(11-12)^. It is opportune to carry out the analysis of undergraduate students’ sexual behavior from a perspective permeated by sociocultural and vulnerability markers.

Young people’s sexual scripts, described in class 4, denote inconsequential sexual behavior, possibly due to poor discernment regarding the risks of acquiring STIs or the pleasure of danger, with an unplanned pregnancy being the greatest concern. It is noticed that immature thinking, present since adolescence, is a stage and, although the basic skills needed to perceive risks are active, the ability to regulate behaviors consistently and with maturity is still incipient^(2)^.

It is added that it is in adolescence that more attention is given to the potential rewards coming from a risky choice than to the consequences of that decision. P14’s speech denotes the vulnerability of young people in the thought that unprotected sex generates greater pleasure, or that the possibility of exposure to risk excites them, being able to stand out without being contaminated, with a false sense of power and invulnerability^(12)^.

Sexuality is a socially learned behavior, in which several scripts guide people in their sexual experiences, bearing in mind that not everything is practicable at all times. Individuals elaborate their actions according to their ability to relate, fantasies, cultural language and social roles played^(11)^. These scripts are structured on three levels that are presented at the subjective level, in the intention of social coexistence and at the cultural level, namely: intrapsychic, such as information on STI prevention; interpersonal, involving sexual practices with or without the use of STI preventive strategies; and those of cultural scenarios, portrayed by the university environment and interpersonal interactions^(11,13)^.

Young people’s sexual scripts are still guided by the fear of an unplanned pregnancy, as explained by P07 and P33, who sometimes resort to emergency methods, such as the morning-after pill. The greater social exposure of women in the case of pregnancy and the belief that the responsibility for contraception and the consequences of pregnancy are female responsibilities, justifying emergency contraception^(14)^. P48’s speech highlights that health care should be shared among people, due to the information they have on the subject and experiences. This statement is essential for young people in the exercise of their sexual practices, because sexuality is understood as a fundamental aspect of the human life cycle involving desires and practices related to pleasure, freedom, feelings and health, where the physical body is just a foundation for the historical and identity construction that involves other constructions, such as individuality, sexual satisfaction and erotic appetite^(11)^.

In this context, the need for cross-cultural care for human beings is inserted, which is impregnated with meanings in its entirety, essential for knowledge, explanation, interpretation and prediction of the phenomenon of “caring” in nursing. Cultural care, in its concepts, meanings, expressions, patterns, processes and structural forms, can be used differently (diversity) or similarly (universality). These modalities are common to any type of culture in the world^(5)^. Cultures have their own care characteristics, developing a generic care practice, and these characteristics are influenced by the world view, language, religion, sociopolitical, educational, economic, theological, ethno-historical and environmental context, interrelated to the social group in which one lives^(5)^.

Regarding the perspective of transculturality and vulnerability determinants, presented in class 3, it is noted that, with fixed sexual partners, mostly connected by affective ties, stable relationships or the formality of marriage, young people tend to stop using of the optional condom or abolish it. This is because they feel safe with their sexual partnerships, in order to adopt condoms only as a strategy to prevent an unplanned pregnancy. In these relationships, there is an affective-loving bond and an expectation of trust in marriage safety. STI prevention has complex and multifaceted aspects. Cultural, affective and behavioral factors, such as excessive trust in the partner, have a strong influence on condom disuse behaviors^(15-17)^. The use of condoms is related to the availability and accessibility of this resource, and does not require a prescription, providing low-cost and highly effective protection against STIs and unplanned pregnancy. The reduction in the use of this resource in relationships with fixed partnerships can be justified by the immaturity and impulsiveness of young people and/or by their greater age, which corroborate with the feeling of security, due to the use of another contraceptive method by the partner or due to the existence of a more solid relationship^(14,18)^.

Some described the condom as an accessory resource necessary only to prevent an unplanned pregnancy. The condom is widely recognized for the primary prevention of STIs. Young people tend to use it as a risk management strategy, depending on the situation experienced, such as an unplanned pregnancy. Among men, condoms have been more frequently used in casual and homoaffective sex. Among women, the HIV test has been used more as a way to compensate for unprotected sexual intercourse^(18)^.

In class 2, gender defined the roles between men and women in the exercise of sexual practices. Sexual behaviors were permeated by male vulnerabilities, since it is their responsibility to define whether or not the condom would be used, due to the submissive posture adopted by many women, portrayed in the domination of women’s bodies and sexual behavior. Therefore, a strong influence of the historical context and social roles in affective and sexual relationships is perceived^(19)^. Gender roles are taught from childhood and establish the differences between girls and boys. Female and male behaviors are encouraged and reinforced by parents, social institutions and close people in a process of domination of bodies and human sexuality^(4,11)^. This domination is attributed to sociocultural and conditioning behaviors of men over women, such as that carrying the condom is the responsibility of men, in addition to the decision-making power in wanting to use this resource or not.

Men feel more vulnerable in relation to women, considering the multiple justifications and the greater number of sexual partners. Sexual behaviors are differentiated according to gender, and a man’s greater sexual experience is expected and valued. As for women, a more discreet behavior, less sexual exposure, is not “approved” of a sexual behavior similar to that of men.

Religion has influenced human behavior since the dawn of humanity, inducing habits and customs in all cultures. The sexuality and sexual behavior of humanity, in the past, were determined by religion, and many of these determinations are present in the morality of the present day. Sexuality is still structured around values and beliefs that determine what is prohibited and what must be protected, varying according to culture^(19)^.

Social markers are intersectional, because many of them are inseparable, such as gender, ethnicity and social class. When analyzing a person socially, one must consider all social markers and how they are articulated^(6)^. Culture must be conceived as a social production that is born from the confrontation of the intersectionality of social determinants, manifested by social, transcultural behaviors, in the combination of individual and collective belief systems^(4-5)^. There are also behaviors that portray the emancipatory movement and female sexual freedom, which are associated with the increase of their vulnerabilities with the adoption of RSB, such as casual sexual practices and multiple partnerships. Added to this is the fact that women aged between 15 and 49 years did not identify their real risk level for STIs^(2)^.

The family, although influenced by means of control, such as religion, economy, media and culture, has unique value for the socio-historical structuring of men’s and women’s role in society. The family context has a preponderant role in the cultural aspect, acting as a controlling institution that imposes a certain behavior on the members. Information about right and wrong, acceptable or not, is initiated and shaped in this environment, which must include sexual practices and STI preventive strategies^(20)^.

Negotiating the use of condoms was permeated by numerous vulnerabilities, such as the use of alcohol and other drugs, which strongly influenced sexual behavior. The adoption of RSB with exposure to STIs is not uncommon, even among young people with greater access to information, such as undergraduate students^(21)^. Other factors affect the recurrence of sexual practices without using a condom. Among the justifications, there is the woman’s fear of judgment if she is carrying a condom and suggests using it in sexual practices, which is perceived as a condition for promiscuity. It is the duty of men and women to have condoms, to increase their negotiating power and/or to impose their use in sexual practices. The results reinforce the existence of a power relationship between men and women, strong control by men and women with low negotiating power and a position of submission. A study confirms these findings, stating that the negotiation of condom use was carried out by only 31.65% of participants, in addition to the low compliance with continuous use of condoms, favoring exposure to STIs. In affective/sexual relationships, many women are culturally silenced and do not have negotiating power, as can be seen in relation to the (dis)use of condoms^(21)^.

In order to understand the sexual behavior of undergraduate students, it is necessary to understand the sociocultural context where they emerged, that is, to analyze and reflect on the social markers and cross-cultural aspects of the adopted sexual behaviors. The commitment to sex education is urgent and involves not only health care, but also universities and society, in order to outline the consequences that may result from RSB^(3)^. They should include the analysis of their sexual scripts and lifestyles of young people as well as their vulnerabilities^(15)^.

A rereading of sexual behavior is necessary, from the perspective of the Sunrise Model and undergraduate students’ social markers. Thus, social structures and cultural accommodation of how to deal with the health/disease process influence care patterns, their expressions, decisions and professional actions (preservation, maintenance, accommodation and negotiation), through the repatterning and restructuring of care, adding coherence to cultural care^(5)^. Sexuality needs to be understood as a sociocultural production, in which the ways of experiencing sexual pleasures and desires are not provided by nature, but there is a complex combination of meanings and attributions that effectively constitute them. Sexual individuality is, therefore, a set resulting from the development of historical and sociocultural factors, exercised through myths, taboos and power relations. Sexual practices are conducted according to the interpersonal relationships experienced in each stage of life cycle^(11)^.

The transposition of some concepts^(4-5)^ encompassed: a) Culture: set of values, beliefs, norms and life practices of undergraduate students, learned, shared and transmitted, which guide their ways of thinking and sexual practices in a standardized way; b) Worldview: way in which undergraduate students perceive the world around them; c) Social structure: structural/organizational factors related to the university and its determinants, which add culturally congruent meaning to young people’s experiences; d) Environmental context: totality of experiences that give meaning to the university phase. Some metaparadigms^(4-5)^, when transposed, made it possible to deduce the concepts: person: individual between 18-29 years old susceptible to the adoption of RSB and contamination by STIs, culturally inserted in HEIs; environment: sociocultural dimension of the HEI permeated by vulnerabilities that influence the adoption of RSB.

In carrying out cross-cultural care^(4-5)^, preservation/maintenance is one of the care practices already practiced by the person, which is beneficial or innocuous to their health. Thus, the objective is to identify and encourage the maintenance of safe sexual behavior by undergraduate students. Accommodation and negotiation are actions and decisions to assist, support and facilitate people to adapt with healthcare providers. It is expected that sexual behaviors can be remodeled from the understanding of the sociocultural context, in order to recognize themselves as sexually vulnerable and learn to accommodate their sexual practices to negotiate RSB, in order to eliminate them. Repatterning and restructuring are actions and decisions that aim to facilitate and support the reordering, exchange or modification of ways of life, to benefit from available health care standards. Undergraduate students would receive interventions and professional care to encourage autonomy by replacing RSB with safe sexual behavior, managing vulnerabilities, raising awareness and mobilizing these young people.

The perspective of culturally congruent care with educational, political, legal, technological and economic dimensions needs to consider the sociocultural structure^(5)^ and the reality of HEIs. The university environment was recognized as favorable to the adoption of RSB. This collective perception is justified by: technological, religious, social, cultural, lifestyle and educational dimension, whose accommodations require the remodeling of lifestyles; insertion of assistive technologies; public policies that make the university environment safe; adequacy of HEIs and their professors for the construction and effective viability of the perspective that the university is recognized as an institution that promotes health^(4,22-23)^.

### Study limitations

A possible limitation of this study is associated with the fact that it did not pay attention to the areas of knowledge in students’ training. Thus, other investigations could analyze sexual behavior of undergraduate students, from the perspective of the area of knowledge of each subgroup of students, to verify if there are distinctions.

### Contributions to nursing

The investigation collaborates with the professional practice of nursing, by presenting an existing gap in the care of this group, identifying components of vulnerabilities in the profile of undergraduate students that can be preserved/maintained, accommodated/negotiated and repatterned/structured in the planning of care with cultural congruence, following the elements, concepts and metaparadigms guided by the theoretical-philosophical framework of nursing.

## FINAL CONSIDERATIONS

Undergraduate students, in their sexual practices, tend to adopt RSB associated with social and cross-cultural markers. The cultural actions of care require interventions on the determinants of sexual behavior to enable patterns and practices of expression of (self) care. In this way, it is possible to preserve/maintain safe sexual conduct, accommodate and negotiate RSB in the repatterning/restructuring of safer sexual practices for the prevention of STIs among undergraduate students.

Unsafe sexual behaviors adopted by undergraduate students are associated with social markers that determine sexual behavior in an intersectional and cross-cultural way. This epidemiological, educational and health problem related to the occurrence of STIs, resulting from the multiple vulnerabilities involved in undergraduate students’ sexual practices, could be reduced if HEIs, as institutions that promote health, developed problematized, attractive and engaging educational actions to build knowledge about STIs and preventive practices that could convert experiences/experiences into useful knowledge, capable of remodeling undergraduate students’ sexual behavior.
